# Evaluation of clinical efficacy and safety of cervical trauma collars: differences in immobilization, effect on jugular venous pressure and patient comfort

**DOI:** 10.1186/1757-7241-22-37

**Published:** 2014-06-06

**Authors:** Sigurbergur Karason, Kristbjorn Reynisson, Kristinn Sigvaldason, Gisli H Sigurdsson

**Affiliations:** 1 Department of Anesthesia and Intensive Care, Landspitali University Hospital, Reykjavik, Iceland; 2 Faculty of Medicine, University of Iceland, Reykjavik, Iceland; 3 Department of Radiology, Landspitali University Hospital, Reykjavik, Iceland

**Keywords:** Cervical collar, Intracranial pressure, Cervical spine injury, Head trauma, Internal jugular venous pressure

## Abstract

**Background:**

Concern has been raised that cervical collars may increase intracranial pressure in traumatic brain injury. The purpose of this study was to compare four types of cervical collars regarding efficacy of immobilizing the neck, effect on jugular venous pressure (JVP), as a surrogate for possible effect on intracranial pressure, and patient comfort in healthy volunteers.

**Methods:**

The characteristics of four widely used cervical collars (Laerdal Stifneck® (SN), Vista® (VI), Miami J Advanced® (MJ), Philadelphia® (PH)) were studied in ten volunteers. Neck movement was measured with goniometry, JVP was measured directly through an endovascular catheter and participants graded the collars according to comfort on a scale 1–5.

**Results:**

The mean age of participants was 27 ± 5 yr and BMI 26 ± 5. The mean neck movement (53 ± 9°) decreased significantly with all the collars (p < 0.001) from 18 ± 7° to 25 ± 9° (SN < MJ < PH < VI). There was a significant increase in mean JVP (9.4 ± 1.4 mmHg) with three of the collars, but not with SN, from 10.5 ± 2.1 mmHg to 16.3 ± 3.3 mmHg (SN < MJ < VI < PH). The grade of comfort between collars varied from 4.2 ± 0.8 to 2.2 ± 0.8 (VI > MJ > SN > PH).

**Conclusion:**

Stifneck and Miami J collars offered the most efficient immobilization of the neck with the least effect on JVP. Vista and Miami J were the most comfortable ones. The methodology used in this study may offer a new approach to evaluate clinical efficacy and safety of neck collars and aid their continued development.

## Background

Cervical spine immobilization with a cervical collar is a routine procedure during extrication, transport and initial evaluation of trauma victims or until clearance of cervical spine injury has been made [1]. If cervical spine injury is confirmed, the collar is kept on until surgery and even in some cases as treatment for several weeks if surgery is not considered necessary [2,3].

The purpose of routine use of cervical collars in severe trauma victims is to reduce the risk of secondary damage to the spinal cord. It has been shown that among severely injured, unconscious and intubated blunt trauma patients 14% have cervical injury and 7% being unstable [4]. Since clinical assessment of the cervical spine is limited in the unconscious patients, reliable immobilization of the neck has been considered imperative in this patient group. On the other hand critics have pointed out lack of randomized controlled trials that confirm clinical benefits of spinal immobilization after trauma and the potential risk of complications with the use of neck collars [5]. Consequently, it should be considered of great importance to develop neck collars that provide secure immobilization while minimizing possible adverse effects.

**Figure 1:**
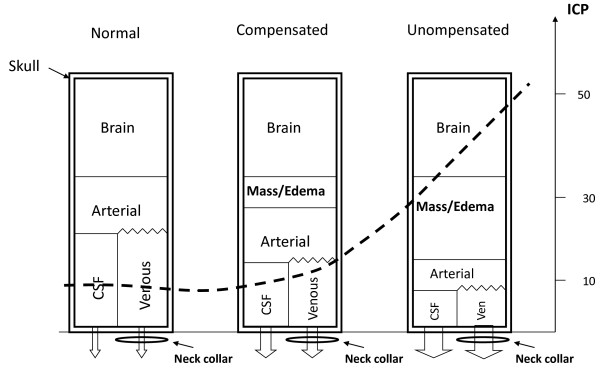
**The relationship between intracranial volumes and intracranial pressures (ICP).** The figure shows the different compartments within the skull, and what happens to them and the ICP (broken line) when a new pathological compartment appears. As volume increases inside the skull compensation may occur up to a certain limit by decreasing cerebrospinal fluid (CSF) and venous blood inside it. A neck collar may obstruct venous outflow, hampering this mechanism and causing a move to the right on the ICP curve. How much ICP will increase will depend on where on the pressure curve the patient is positioned.

It has been shown that different types of cervical collars immobilize the cervical spine variably well [6-10]. Also, concern has been raised that neck collars may increase intracranial pressure (ICP) [11] in patients with head injury by hindering venous outflow through neck veins, acting as a tourniquet around the neck, adding to intracranial blood volume and pressure (Figure 1) [12]. The effect of a rigid neck collar on ICP has been studied in head trauma patients with direct intracranial pressure measurement showing a mean increase of 4.5 mmHg [13-15]. Since it is difficult to perform comparative studies on such a vulnerable population as trauma victims, risking increase of ICP to dangerous levels, surrogate markers have been sought. In healthy subjects a 37% increase in the cross-sectional area of the internal jugular vein, measured by ultrasound, has been shown after application of a cervical collar thought to reflect increased jugular vein pressure [16]. However, measurement with ultrasound is technically difficult as most collars do not allow access of an ultrasound probe to the area over the jugular veins, and, even so, just the pressure exerted by the probe on the skin above the jugular vein may influence the results of measurements.

There are also several other adverse effects of cervical spine immobilization known, such as increase in respiratory effort, skin ischemia, pain and discomfort [5,17]. Cervical collars are experienced as being variably comfortable to wear, which seems to be mainly attributable to the amount of pressure the collars exert on the skin [18].

According to above it has been shown that biomechanical qualities of different types of neck collars vary in regards to immobilization and comfort. We hypothesized that this would also be the case in influence on jugular venous pressure.

The purpose of this study was to compare four different types of neck collars frequently used in acute trauma care regarding 1) efficacy in immobilizing the neck, 2) effect on internal jugular venous pressure, as a surrogate for possible effect on ICP and 3) patient comfort.

## Methods

This was a joint research project between Landspitali University Hospital, Reykjavík, Iceland, and Össur Inc., Reykjavik, Iceland, an international prosthetic company. After securing approval and a written agreement from both parties and the National Bioethics Committee (no. 11-039-S1), 10 healthy adult volunteers gave their written consent for participation in the study after responding to an advertisement and being screened for a clean bill of health (ASA I).

Four types of collars were chosen for the study, all widely used in acute trauma care, Laerdal Stifneck® Select™ Collars-Adult (Laerdal Medical AS, Stavanger, Norway), Philadelphia® Tracheotomy Collar (Össur, Reykjavik, Iceland), Miami J® Advanced (Össur, Reykjavik, Iceland) and Vista® Collar (Aspen Medical Products Inc., Irvine, California, USA).

During all measurements the collars were applied by a single certified orthotist, who was blinded to results. The orthotist decided before any measurements the appropriate size and settings of the collars for each individual, which were then used during all measurements. Randomization of collars for each individual took place by drawing from an envelope before each measurement session. Measurement of immobilization and grading of comfort took place at Össur Inc, while measurement of jugular venous pressure took place at Landspitali University Hospital and those involved at each location where blinded for each other results. The statistical analysis, interpretation of results and writing of a manuscript where solely executed by the participants from Landspitali University Hospital.

### Immobilization

The degree of flexion, extension, lateral tilt to right and left and rotation of the neck to right and left without a collar and with the four different types of collars put on in random order were measured in the sitting position with voluntary movement, using goniometric technique (CROM Deluxe, Performance Attainment Associates, 12805 Lake Blvd Lindstrom, Minnesota, USA). Each movement was measured three times. To simplify the presentation of results, the average movement for all directions was calculated and used for comparisons.

### Effect on internal jugular venous pressure

Jugular venous pressure measurements were performed with a micro-catheter with several side holes at its end (Beacon® Tip Royal Flush® Plus High-Flow Catheter, William Cook, Europe ApS, Sandet 6 DK 4632 Bjaeverskov, Denmark). It was inserted by a specialist in invasive radiology through veins at the right cubital fossa into the internal jugular vein, either right or left, under fluoroscopy. After placing the catheter tip just under the base of the skull the position was verified by two X-ray pictures with a 90-degree difference in view. The catheter was then connected to a continuous flush pressure transducer for intravascular pressure measurements (Gabarith™ PMSET 1DT-XX, Becton Dickinson Critical Care Systems Pte Ltd, 198 Yishun Ave. 7, Singapore). The transducer was connected to a multi-module monitor for display of continuous invasive pressure (HP Agilent Critical/Cardiac Care monitor). The pressure transducer was positioned on the upper arm of the patient at the height of the heart during measurements.

Baseline measurements without a neck collar were performed in the supine position then moved in a 20-degree anti-Trendelenburg position (head up) and registered again after 30 seconds or later if it took longer for the pressure curve to stabilize. This was repeated twice. Then the four types of neck collars were fitted on the patients in a random order and the pressure measurements repeated, as described above, twice with each collar in supine and 20-degree anti-Trendelenburg position, or four measurements altogether. After finishing all the collar measurements, the baseline measurements were repeated without any collar.

### Comfort

After the goniometric measurements the participants graded the collars according to how comfortable they were to wear, using a scale from one to five, five being the most comfortable score.

### Sample size

Our main interest was to study the effect of the different collars on jugular venous pressure. To our knowledge there have not been any such studies performed with direct pressure measurements in the jugular vein. There are two studies of the effect of a Stifneck collar on ICP in patients with severe head injury with ICP <20 mmHg, which is below what is considered pathological, showing a significant rise in mean intracranial pressure, with standard deviation of 4.1 ± 3.6 mmHg (p < 0.001) [13] and 4.6 ± 3.1 mmHg (p < 0.0001) [13,14]. This would require a sample size of 9 and 14 participants, respectively, when assuming a power of 80% and significance of 5%. However, as explained above, an increase in jugular venous pressure might go unnoticed when measuring intracranial pressure because of compensation mechanisms and could therefore have happened more frequently. Hence, 10 participants were considered a sufficient number for this study.

There has also been one study on the effect of a rigid cervical collar (Ambu Perfit ACE adjustable extrication collar, Ambu Inc.) on internal jugular vein dimensions in healthy volunteers, showing a mean increase and standard deviation of 0.19 ± 0.32 cm^2^[16]. This would indicate the need of a sample size of 17 participants when assuming a power of 80% and significance of 5%. However, as explained above, we consider this a much less accurate method of estimating the effect on jugular venous pressure than direct pressure measurement, so a smaller number should be sufficient. Calculation of sample size was performed with the statistical program R (The R Foundation for Statistical Computing).

### Statistics

Statistical analysis was performed with the statistical program R (The R Foundation for Statistical Computing); values are given as mean ± standard deviation (SD) and minimal (min) and highest (max) value when appropriate. To grade immobilization, a mean value for all range of movements without a collar and for the different collars was calculated and used for comparisons. To grade the effect on pressure, mean values without a collar and with the different collars were calculated and used for comparisons. For ranking comfort a mean value of the grades for the different collars was calculated and used for comparisons.

To study differences between baseline and the different collars regarding immobilization and effect on jugular vein pressure, repeated measures analysis of variance was used. To study differences between grades of comfort of the various collars, a Friedman test was used to confirm differences and then a Wilcoxon test was used to compare them. A p value < 0.05 was considered statistically significant.

## Results

### Participants

Ten volunteers participated, 5 men and 5 women. Their mean age was 27.4 ± 5.5 yr (min 21, max 28), height 176.2 ± 10.5 cm (min 156, max 189), weight 80 ± 9.1 kg (min 60, max 92) and BMI 26 ± 4.5 (min 20.3, max 35.3). None of them suffered any complications during the study.

### Immobilization

The mean degree of neck movement decreased significantly from baseline (53 ± 9°) with all the collars (p < 0.001) but to a variable extent with the following values: Stifneck 18 ± 7°, Miami J 21 ± 10°, Philadelphia 22 ± 8° and Vista 25 ± 9°. Significance of degree of immobilization between collars is shown in Table 1.

**Table 1:** Immobilization

			**P-values of comparisons**				
**Collar type**	**Mean°**	**Range°**	**Without**	**Stifneck**	**Miami J**	**Philadelphia**	**Vista**
Without	53 ± 9	45 - 65					
Stifneck	18 ± 7	12 - 29	< 0.001				
Miami J	21 ± 10	13 - 38	< 0.001	0.06			
Philadelphia	22 ± 8	15 - 32	< 0.001	0.01	0.98		
Vista	25 ± 9	16 - 39	< 0.001	< 0.001	0.004	0.027	-

The mean, standard deviation and range of movement at baseline (without a collar) and with the four different collars tested. Collars in order of decreasing immobilization. P-values of comparisons are shown in the right part of the table.

### Effect on internal jugular venous pressure

**Figure 2:**
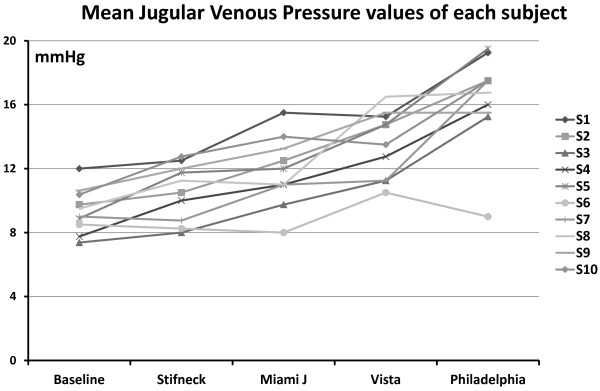
The mean jugular venous pressure values of each individual subject (S) at baseline (without a collar) and with the various cervical collars.

There was not a statistically significant difference in jugular vein pressure without a neck collar before or after measurements with the various collars. Neither was there a significant difference between pressure measurements, with or without collars, at supine position or in a 20-degree anti-Trendelenburg position. Therefore, all four measurements (supine ×2 and 20-degree anti-Trendelenburg ×2) for each collar and all eight baseline measurements (four before and four after wearing the collars) were averaged and used for comparisons. All the collars caused an increase in the average jugular venous pressure from baseline (9.4 ± 1.4 mmHg) but to a variable degree, with the following values: Stifneck (10.5 ± 2.1 mmHg), Miami J (11.7 ± 2.4 mmHg), Vista (13.5 ± 2.5 mmHg) and Philadelphia (16.3 ± 3.3 mmHg). The mean jugular venous pressure value for every subject at each measurement point are shown in Figure 2 and the significance between collars regarding the effect on jugular venous pressure is shown in Table 2.

**Table 2:** Jugular venous pressure

			**P-values of comparisons**				
**Collar type**	**Mean mmHg**	**Range mmHg**	**Without**	**Stifneck**	**Miami J**	**Vista**	**Philadelphia**
Without	9.4 ± 1.4	6 – 13.5					
Stifneck	10.5 ± 2.1	5.5 - 14	0,146				
Miami J	11.7 ± 2.4	5 - 16	< 0.001	0.269			
Vista	13.5 ± 2.5	10 – 17.5	< 0.001	< 0.001	0.024		
Philadelphia	16.3 ± 3.3	7.5 - 20	< 0.001	< 0.001	< 0.001	< 0.001	-

The mean, standard deviation and range of jugular venous pressure, at baseline (without a collar) and with the four different collars tested. Collars in order of increasing effect on jugular venous pressure. P-values of comparisons are shown in the right part of the table.

### Comfort

Vista received the highest grade of comfort by the participants (4.2 ± 0.8), followed by Miami J (3.9 ± 1.0), Stifneck (2.8 ± 1.0) and Philadelphia (2.2 ± 0.8). The significance of comfort between the various collars is shown in Table 3.

**Table 3:** Comfort

			**P-values of comparisons**			
**Collar type**	**Mean degree of comfort**	**Range of comfort**	**Vista**	**Miami J**	**Stifneck**	**Philadelphia**
Vista	4.2 ± 0.8	3 - 5				
Miami J	3.9 ± 1.0	2 - 5	0.55			
Stifneck	2.8 ± 1.0	3 - 5	0.007	0.031		
Philadelphia	2.2 ± 0.8	1 - 3	< 0.001	0.002	0.27	-

The mean, standard deviation and range of comfort (scale 1-5) of the various collars as experienced by the participants. Collars in order of decreasing comfort. P-values of comparisons are shown to the right.

## Discussion

In this study of four widely used cervical collars their biomechanical qualities varied in efficacy of immobilizing the neck, to what degree they increased internal jugular venous pressure and how comfortable they were experienced to wear. All to an extent, that might have clinical importance.

The average age of the 10 participants was just under 30 years, and their average BMI (26 ± 4.5) is at the lower range of what is considered overweight. However, there was quite a difference of demographic parameters within the group, as seen by the standard deviations and the minimal and maximal values. Despite these biometrical variations of the participants, the physiological changes caused by the different collars were fairly consistent, indicating that the biomechanical property of each type of neck collar is quite distinct.

**Figure 3:**
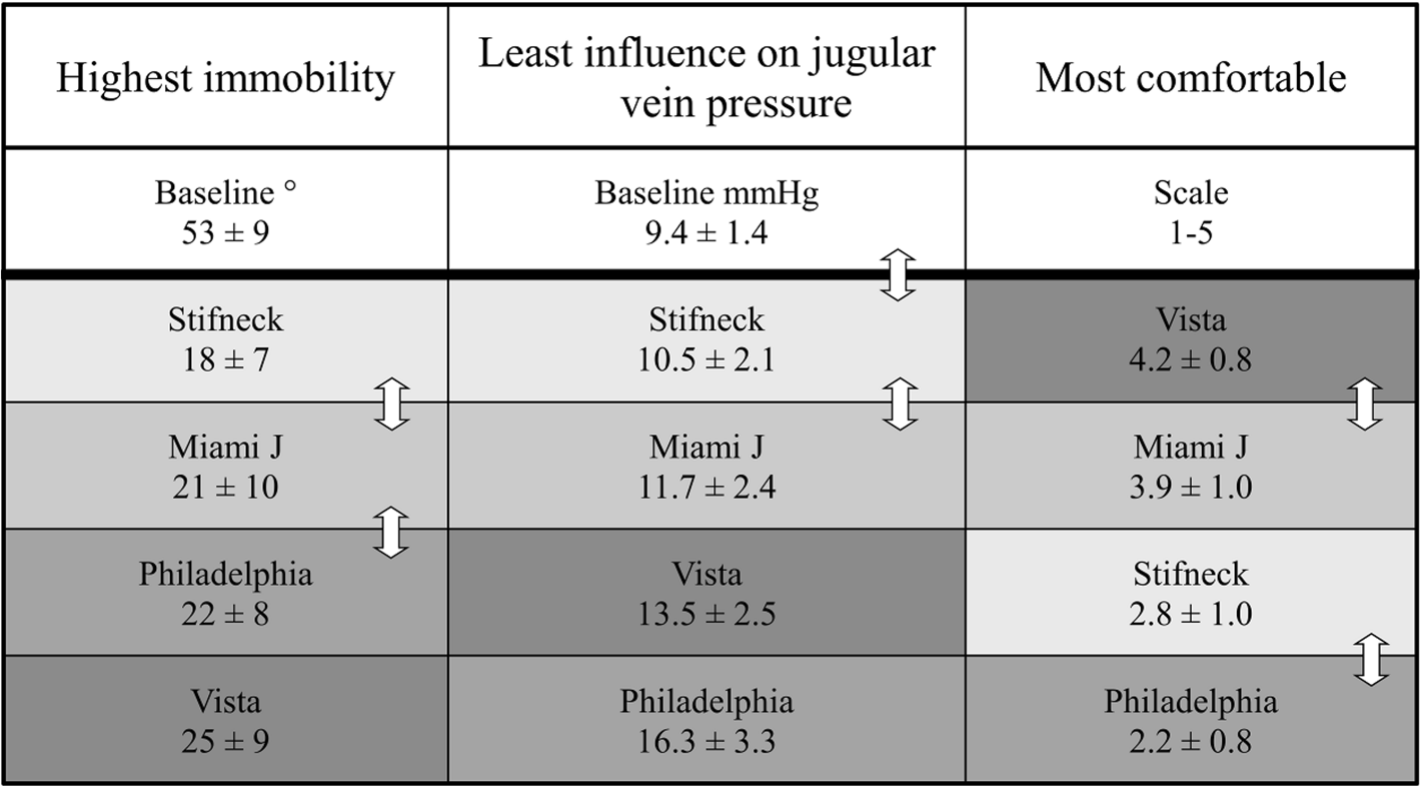
**Summary of results.** Collars in order of decreasing immobilization, increasing effect on jugular venous pressure and decreasing comfort. Tables 1, 2 and 3 show where there was a significant difference between the collars, compared to baseline and each other. However, in this figure a double-headed arrow is used to show where there was not a significant difference between entities. The Stifneck collar seems to suit well in emergency circumstances (greatest immobilization, least effect on jugular venous pressure, but third regarding comfort, so it should probably not be used for long-term treatment). The Miami J collar seems to be a good all-round collar for both emergency and long-term treatment (second in all parameters without differing significantly from those in the first place). The Vista collar seems more suited for long-term treatment (first place regarding comfort, third regarding effect on jugular venous pressure but forth concerning immobilization). The Philadelphia collar seems only suited for short-term treatment (highest effect on jugular venous pressures, third concerning immobilization and fourth place regarding comfort).

The degree of neck movement was significantly reduced from baseline with all the four collars, but to a various degree, as movement decreased from 53° to between 18° and 25° (Figure 3). It is difficult to compare these results with other studies as different methods are used to measure immobilization and present outcomes. However, the total angular displacement for Stifneck was reported to be 17.55 ± 8.9° in one study [19], which is very similar to our results of 18 ± 7°, indicating a reliable setup of measurements. It is intricate to judge the clinical importance of the extent of immobilization in patients with an unstable cervical spine injury. Also, for simplicity of presentation we present only average movement for all directions which might devalue important differences between devices, but our main interest was to study the effect on jugular venous pressure. There are several other studies that have compared the relative effectiveness of preventing spinal motion of various types of neck collars [6-10].

The baseline jugular venous pressure (9.4 ± 1.4 mmHg) increased with all of collars but significantly with only three of them, and not with Stifneck, or from 10.5 ± 2.1 mmHg to 16.3 ± 3.3 mmHg (Figure 3). Even though the Stifneck collar had the least effect on jugular venous pressure in our study on healthy volunteers, it has been shown to increase ICP on average by 4.5 mmHg in a study on head trauma patients, where ICP was measured directly, [14] emphasizing how vulnerable such patients can be.

During pressure measurements the pressure transducer was attached to the upper arm, at the height of the heart, to mimic classical central venous pressure measurements. It was considered of greater interest to measure the difference in jugular venous pressure with and without the different collars and measure what effect changes in position would have on it, rather than attempting to measure the actual pressure value in the jugular vein and positioning the pressure transducer at the height of the neck (the tip of the catheter). In the supine position, zero degrees, the height of the pressure transducer will not make any difference, but in the 20-degree anti-Trendelenburg position the value will increase due to the height of the fluid column from the neck to the transducer but decrease because of shift of the venous blood pool in direction of the legs due to gravity. As stated in the results, there was not a significant difference between the jugular pressure measurements without or with collars in the supine and the 20-degree anti-Trendelenburg position, indicating that the amount of pressure propagated in the direction of the skull, through the veins, is similar in both cases. However, a 30-degree elevation of the head of the bed is recommended in treatment of intracranial hypertension of both adults and children, to optimize cerebral venous outflow [20,21].

The increase in ICP that neck collars may produce is believed to be caused by obstruction of venous outflow from the skull (Figure 1). It is however not known to what extent jugular venous pressure is propagated into the skull [22-25]. Furthermore, how much effect jugular venous pressure will have on ICP will also depend on where on the volume/pressure curve the individual is positioned. If a person is positioned on the left end on the pressure/volume curve, no or little effect will be seen, but if on the right end, a sharp rise in ICP may result, with possible deleterious effects on cerebral blood flow (Figure 1). To our knowledge no other reports have been published describing comparable methods of studying cervical collars as was done in the present study.

The participants found the comfort of wearing the collars varied (Figure 3). It is recognized that the comfort of wearing collars varies, and that they exert different amounts of pressure on the skin. In one study comparing four types of collars, Stifneck and Philadelphia caused the greatest pressure on skin and were also experienced to be the most uncomfortable, [18] while Miami J caused the least pressure on skin and was experienced as the most comfortable one. There may therefore be a correlation between experienced discomfort and exerted skin pressure.

The main limitation of this study is that the participants were healthy volunteers without direct ICP measurement and not patients with severe head injury. However, for developmental purposes it is not practically possible to perform measurements on such patients. The number of participants in this study was determined according to previous studies and even though it was rather small, fairly distinct characteristics were identified for each collar, indicating distinct biomechanical properties of each collar type and reliable measurement methods. A single certified orthotist determined the size and settings of the different collars for all participants and also fitted them to the participants, standardizing this process as far as possible. We asked the patients to grade the collars according to comfort although it may have been more objective to perform direct measurements of pressure exerted by the different collars on the skin and further evaluate the risk of causing pressure sores. However, there appears to be a close correlation between the amount of skin pressure exerted by the different collars and comfort/discomfort experienced by the wearer [18]. Finally, firm measures were taken to minimize influence from industry as the jugular venous pressure measurements, the main objective of the study, as well as evaluation and presentation of results and writing of the manuscript, were solely performed by the authors, that have no connection to or commercial interests in Össur Inc.

In conclusion, the four types of collars tested in this study showed rather distinct biomechanical properties, despite quite a range of demographic parameters among participants, indicating reliable measurement methods. The methodology used in this study – determining the degree of immobilization provided by the various collars, measuring directly their influence on internal jugular venous pressure and grading their level of comfort – could be a new approach to evaluate the clinical efficacy and safety of neck collars, in order to advance their continued development.

## Competing interests

The study was supported by an unconditional grant from Össur Inc. which also offered expert assistance from their Research and Development Unit. Employees of Össur Inc. did not participate in evaluation of the results or in writing this manuscript. The authors are all employees of Landspitali University Hospital with no connection to Össur Inc. No other conflicts of interests are declared.
